# Overexpression of Regulatory T Cells Type 1 (Tr1) Specific Markers in a Patient with HCV-Induced Hepatocellular Carcinoma

**DOI:** 10.1155/2013/928485

**Published:** 2013-10-23

**Authors:** Laurissa Ouaguia, Olivier Morales, Dhafer Mrizak, Khaldoun Ghazal, Emmanuel Boleslawski, Claude Auriault, Véronique Pancré, Yvan de Launoit, Filoména Conti, Nadira Delhem

**Affiliations:** ^1^CNRS UMR 8161, Institut de Biologie de Lille, 1 rue du Professeur Calmette, 59021 Lille Cedex, France; ^2^UPMC Université Paris 6, Inserm, UMR-S938, Centre de Recherche Saint Antoine, 75012 Paris, France; ^3^Service de Chirurgie Digestive et Transplantations, Hôpital Huriez, CHU, Université Nord-de-France, 59000 Lille, France; ^4^Centre de Transplantation Hépatique, Pôle Digestif, APHP, Hôpital Saint Antoine, 184 rue de Faubourg Saint Antoine, 75012 Paris, France

## Abstract

Hepatitis C virus (HCV) is an important causative agent of liver disease, but factors that determine the resolution or progression of infection are poorly understood. In this study, we suggested that existence of immunosuppressive mechanisms, supported by regulatory T cells and especially the regulatory T cell 1 subset (Tr1), may explain the impaired immune response during infection and thus the fibrosis aggravation to hepatocellular carcinoma (HCC). Using quantitative real-time PCR, we investigated the intra-hepatic presence of Tr1 cells in biopsies from a genotype 1b infected patient followed for an 18-year period from cirrhosis to HCC. We described a significant increase of gene expression in particular for the cytokines IL-10, TGF-*β*, and their receptors that were perfectly correlated with an increased expression of the Tr1 specific markers (combined expression of CD4, CD18, and CD49b). This was strongly marked since the patient evolved in the pathology and could explain the failure of the treatment. In conclusion, evidence of regulatory T cell installation in the liver of chronically infected patient with cirrhosis and HCC suggests for the first time a key role for these cells in the course of HCV infection.

## 1. Introduction

Hepatitis C virus (HCV) is a parentally transmitted hepatotropic RNA virus that causes chronic hepatitis, which may lead to cirrhosis and hepatocellular carcinoma (HCC) [1]. Evidence suggests that clearance and control of HCV infection during acute phase is dependent on vigorous and multispecific CD4 and CD8 T lymphocyte responses [2, 3]. On the contrary, the development and maintenance of chronic infection is linked to weak or absence of HCV-specific Th1 response and to the presence of Th2 cytokines (IL-4 and IL-10) [4, 5]. So, some studies have implicated IL-10 in HCV pathogenesis [4, 6]. Regulatory T cell 1 (Tr1) was first described in 1996 [7, 8] as a further subtype of CD4 T cells, without specific markers but with cytokine profile distinct from that of Th1 or Th2 cells. Indeed, they were shown as secreting high level of IL-10, moderate levels of IFN-*γ* and IL-5, quite undetectable IL-4, and low amounts of transforming growth factor (TGF-*β*). Recent studies have suggested that Tr1 could be induced against bacterial, viral and parasite antigens *in vivo* and might prevent infection-induced immunopathology or prolong pathogen persistence by suppressing protective Th1 response [9, 10] but has never been directly implicated in hepatitis C viral pathogenesis. The dysfunction of the immune response against HCV could be explained by immunosuppressive mechanisms supported by Tr1 via their high production of IL-10. The recent identification of combined expression of CD4, CD18 (integrin *α*2), and CD49b (integrin *β*2) as specific markers for Tr1 cells should facilitate their characterization *in vivo* [11]. An increased frequency of Tregs was recently described in the blood of patients with persistent HCV infection when compared with those who had cleared HCV [12, 13]. It has been proposed that Tregs contribute to HCV persistence by suppressing HCV-specific T-cell responses [14, 15]. Some studies have shown a correlation between a reduced HCV-specific T-cell response and the secretion of IL-10 and TGF-*β* by liver-infiltrating Tregs [12] and that Tregs may inhibit HCV-specific T-cell activity in a cell-cell contact manner [12, 13]. It has also been shown that Treg levels are significantly enhanced in recurrent hepatitis C and that Treg type 1 cell (Tr1) levels are specifically higher in severe recurrent hepatitis C [16]. These findings suggest that Tregs may be implicated in the pathogenesis of HCV recurrence.

Moreover, the opportunity of studying three histologically well-defined liver biopsies of an HCV genotype 1b infected patient followed from the chronic infection to the HCC allowed us to investigate the Tr1 cells infiltration within these liver biospies. The first biopsy was performed during the chronic phase (date not specified in the file) and appeared normal without any liver injury, the second one was performed during the cirrhotic phase (March 1990), and the third one was performed during HCC (1998). Thus, we examined the intrahepatic T cell response in terms of cytokines, cytokine receptors, and adhesion molecules in a patient under IFN-*α*/ribavirin treatment and who remained persistently infected >18 years.

## 2. Materials and Methods

### 2.1. Chronically HCV Infected Patient

The HCV infected patient was followed up in the Hepatogastroenterology Department of the Necker University Hospital. Information concerning the patient included age ALAT > three times the normal and negative anti-HAV, HBv, and HEV IgM, and positive serology for HCV (third generation microparticle EIA (Abbott Axsym, Abbott Park)) confirmed by RIBA strip immunoblot assay (Chiron Corporation, Emeryville, CA). The patient was chronically infected by HCV genotype 1b. Genotyping of HCV was done using a multiplex PCR method with genotype-specific primers [17], and the concentration of HCV RNA was determined by RT-PCR with the Cobas Amplicor HCV monitor test (Roche Diagnostics, Branchburg, NJ). The patient presented no human immunodeficiency virus (HIV) coinfection, immunosuppressive therapy, chronic liver disease due to hepatitis B virus, autoimmune hepatitis, or primary biliary cirrhosis and other causes of chronic liver disease.

### 2.2. Liver Biopsy Samples

Three liver biopsies were performed for one patient who underwent several biopsies for a longitudinal study. The three biopsies were performed as part of the routine medical followup and were obtained by the standard Menghini procedure (needle diameter: 1.6 mm) with a biopsy sample length of approximately 1 cm. Based on histologic fibrosis (*F*) and necroinflammatory (NI) scores, liver biopsies were divided into biopsies with small hepatitis and without liver lesion (*F* < or = 1/6; *N* < or = 1/18), biopsies with fibrosis grade and liver lesions corresponding to histologically proved cirrhosis (*F* = 6/6), and biopsies with HCC histologically proved by anatomopathologic expertise. All biopsies were snap-frozen for subsequent RNA extraction.

The study was approved by the Institut de Biologie de Lille (CNRS) Institutional Review Boards and informed consent was obtained in writing from the donor. 

### 2.3. RNA Extraction

Extraction of total RNA from frozen liver biopsy samples was performed using TRIzol reagent (GIBCO BRL, Invitrogen, Scotland, UK) as described by the manufacturer. Tissue samples were homogenized in 300 *μ*L of TRIzol. Homogenized samples were incubated for 5 minutes at room temperature and resuspended in 60 *μ*L of chloroform (PROLABO, Merck Eurolab, France). Total RNA was then immunoprecipitated with 100 *μ*L of isopropanol (ACROS Organics, USA). The RNA pellet was finally washed twice with 75% ethanol (CARLO ERBA Reagent, France) and dissolved in DNAse, RNAse-free distilled water (GIBCO BRL, Invitrogen, Scotland, UK). Before reverse transcription, we performed DNAse treatment using the kit Message Clean (GenHunter Corp., France).

### 2.4. Light Cycler-Based PCR Assay

cDNA was synthetized from total RNA at a concentration of 100 ng/*μ*L using random hexamers and Superscript reverse transcriptase (GIBCO BRL, Invitrogen, Scotland, UK). The quantification of transcripts from liver samples was performed by real-time quantitative RT-PCR using the Light Cycler system (Roche Diagnostics, Mannheim, Germany). The PCR mixture contained the following: *Taq* polymerase, 1X of Light-Cycler DNA master SYBR Green I (Roche Diagnostics, Meylan, France), 3 mM of MgCl_2_, 0.5 *μ*mol/L of each primer, and 1 *μ*L of cDNA preparation (patient cDNA samples) in a total volume of 20 *μ*L. Thirty-two samples were run in parallel by performing an initial denaturation at 95°C for 8 minutes; the PCR reactions were cycled 35 to 40 times as follows: 15 seconds at 95°C, 7 seconds at the appropriate annealing temperature (Table 1), and 18 to 64 seconds at 72°C according to the length of the target sequence, followed by annealing (40 s at 58°C). Fluorescence intensity was measured at the end of each elongation phase. The melting curve analysis was carried out immediately after amplification, following the manufacturer's instructions. Amplification of liver cDNA was successfully repeated three times with cDNA from the same extraction.

### 2.5. Primers

All primers were designed for real-time PCR use and were purchased from MWG-Biotech (Germany) (cf. Table 1). Housekeeping genes *β*-actin and G3PDH were used as control. Samples are quantified using relative standard curves for each amplification reaction, and results were normalized to the internal controls *β*-actin and G3PDH.

### 2.6. Gel Electrophoresis

After each Light Cycler run, agarose gel electrophoresis with TBE (Tris-borate-EDTA) 1.5% agarose (Sigma-Aldrich, Germany) gel, followed by DNA staining with ethidium bromide, was performed to have an independent validation check of the presence of an amplicon. In order to control the length of the amplicon generated, a 100 bp DNA ladder (GIBCO BRL, Invitrogen, Scotland, UK) was used.

## 3. Results and Discussion

As the lymphotropism of hepatitis C virus (HCV) has already been ascertained, and in the light of the fact that the immune defense system is an organized network composed of functionally immune cells, this study was carried out to verify the possible involvement of Tr1 cells in the progression of HCV-related chronic hepatitis.

**Figure 1:**
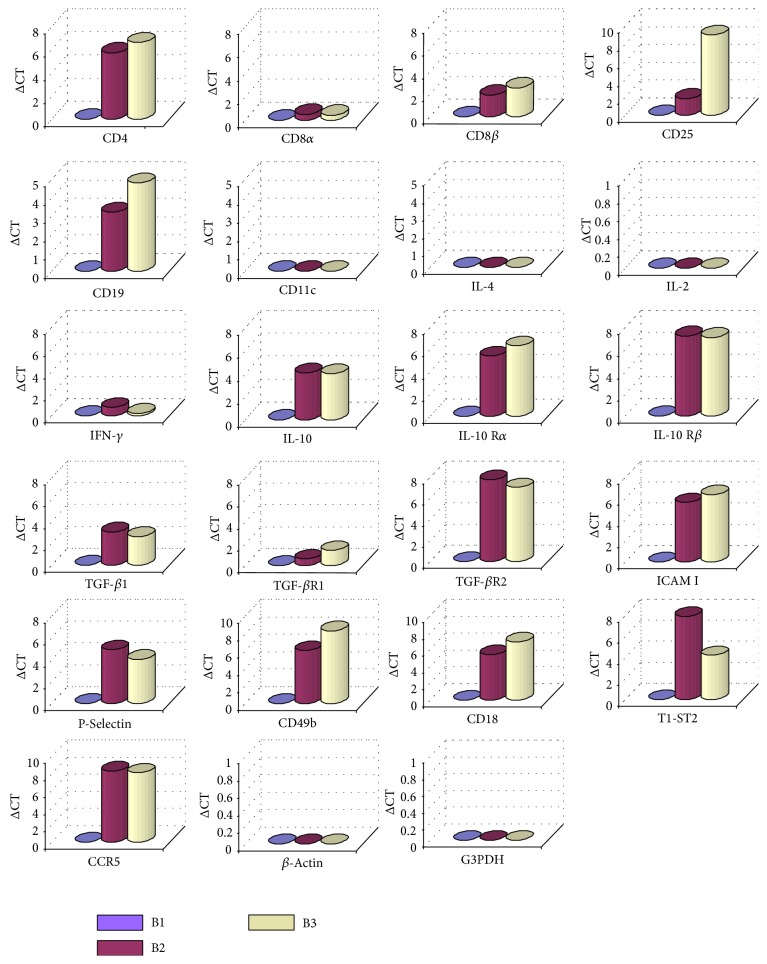
Light Cycler-based PCR assay analysis. Histogram analysis of liver biopsies from the same genotype 1b infected patient for expression of various immune markers. Amplification of liver cDNA was successfully repeated three times with cDNA from the same extraction (B1 = healthy biopsy; B2 = cirrhotic biopsy; B3 = HCC biopsy). The prevalence of gene expression was calculated by comparing the cycle numbers (CT) of the logarithmic linear phase of samples 2 and 3 with the cycle numbers of sample 1, which was designed as reference (ΔCT) Samples are quantified using relative standard curves for each amplification reaction, and results were normalized to the internal controls *β*-actin and G3PDH.

**Figure 2:**
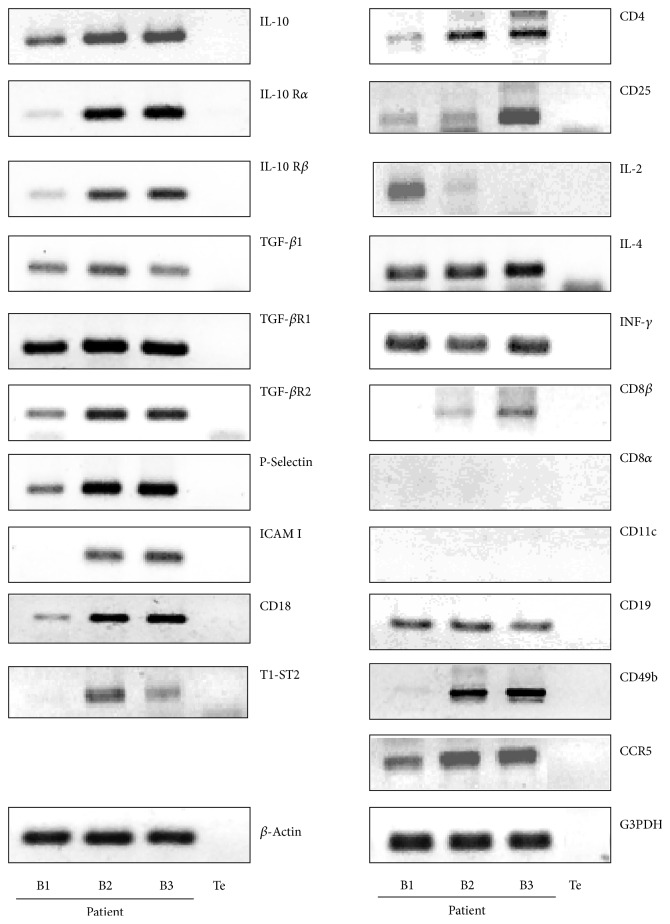
Agarose gel electrophoresis analysis. Agarose gel electrophoresis analysis showed amplification of cDNA extracted from liver biopsies, by the Light Cycler technique for T regulatory markers and immunoregulatory associated factors.

In this study, we first analyzed the quantitative expression of the different intrahepatic cell populations by real-time PCR using Light Cycler system (Figure 1), and we performed after each RT-PCR an agarose gel electrophoresis to have an independent validation check (Figure 2). We observed a significant increase of CD19 expression in cirrhotic and HCC liver biopsies in comparison with the first biopsy without liver lesions. This observation confirmed data of the literature showing disturbances of B lymphocyte activation and function associated with HCV chronic infection [17, 18]. The CD8*β* but not CD8*α* gene expression was also increased in the cirrhotic and HCC biopsies. This observation was in correlation with recent data evidencing by a similar approach of real-time quantitative assays a significant increase and infiltration of CD8*β* during chronic HCV infection [19]. 

But the major observation of our work was the significant increase of CD4 expression in the course of time and proportionally with the severity of the fibrosis. However, we did not observe a variation of IFN-*γ* or IL-2 expression, suggesting that the CD4 marker detected in cirrhosis and HCC liver biopsies was probably not associated with the Th1 protective phenotype. Due to the absence of IL-4 expression, the CD4 cells with Th2 phenotype were also excluded in accordance with the work of Bergamini et al. [20]. A further subtype of CD4 T cells, with immunosuppressive function and cytokine profiles distinct from either Th1 or Th2 T cells, termed regulatory T cells has been described. They include Tr1 cells which secrete high level of IL-10 and low to moderate levels of TGF-*β*, Th3 cells which primarily secrete TGF-*β*, and CD4^+^CD25^+^ cells, which inhibit immune response through cell-cell contact (9). Although the level of IL-10 and TGF-*β* produced *in vitro* may not be significantly higher than that produced by classical Th2 cells, what is important is that Tr1 cells make these cytokines in the absence of significant levels of IL-4 [8]. We confirmed within cirrhotic and HCC biopsies a significant increase of expression of IL-10, TGF-*β*1 and their respective receptors IL-10R*α*, IL-10R*β*, and TGF-*β*R2 whereas we did not detect an increase of IL-4. So, the production of IL-10 and TGF-*β* in the absence of IL-4 argued in favor of the presence of Tr1 in the cirrhotic and HCC biopsies. Although liver dendritic cells also secrete high levels of IL-10, in the present study we could not detect CD11c expression, confirming a Tr1 cells origin for IL-10 production. In this sense, functional alteration of HCV-specific CD4^+^ T cells or failure to develop a long-lasting T helper response was recently correlated to a loss of IFN-*γ* secretion and the presence of a significant antigen-specific IL-10 and TGF-*β*, which could contribute to chronic hepatitis C persistence [21]. Moreover, TGF-*β* plays a pivotal role in inducing fibrosis and has been proposed as its surrogate marker. Indeed, a very interesting study has shown that serum TGF-*β*1 could be used to assess therapeutic outcome and short-term prognosis of HCV-related chronic hepatitis [22].

Moreover, results obtained for Tr1 specific markers CD49b and CD18 confirmed the Tr1 phenotype and showed that Tr1 cells increased during the progression of the pathology. Despite their low proliferative capacity, cloned Tr1 express normal levels of T cell activation markers such as CD25 following TCR-mediated activation. They also expressed CCR5 and T1-ST2, an IL-1-R-like molecule, markers previously expressed preferentially on Th1 and Th2 cells, respectively [9]. We clearly confirmed here within cirrhotic and HCC biopsies the expression of CD25, CCR5, and T1-ST2 in relation to the presence of Tr1 cells. In accordance with this Tr1 phenotype, the expression of TGF-*β*-R2 was also observed. However, we cannot exclude the implication of other regulatory T cell subsets, insofar as we observed a significant increase of CD25, Pselectin, and ICAM I expression, which were known as specific markers for CD4^+^CD25^+^ regulatory T cells. These observations were also conforted by other experiments we performed on liver biopsies of three well-defined cohorts of 45 HCV genotype 1b infected patients, including patients without liver lesions, patients with cirrhosis, and patients with histologically proved HCC that confirmed an increase of Tr1 proportional to the aggravation of the pathology [23]. A first implication of regulatory T cells during HCV infection was examined in peripheral blood of patients infected with HCV genotype 1b, showing a secretion of IFN-*γ* or IL-10 but not IL-4 by HCV core-specific CD4 T cell clones [24]. This work demonstrated that helper type 1 and regulatory T cells were induced probably against the same epitopes on the core protein. Moreover, Sugimoto et al. [25] have suggested for the first time that HCV persistence was associated with a reversible CD4-mediated suppression of HCV-specific CD8 T cells and with higher frequency of CD4^+^CD25^+^ regulatory T cells that could directly suppress HCV-specific type 1 CD8 T cells *ex vivo*. Indeed, it is possible that the induction of regulatory T cells during HCV infection may suppress antiviral Th1 and consequently CD8 CTL responses *in vivo*, and this may explain the persistence of infection and prevalence of cirrhosis.

## 4. Conclusion

Evidence of regulatory T cells installation in the liver of a chronically infected patient with cirrhosis and hepatocellular carcinoma (HCC) suggests a key role for these cells in the aggravation of the liver pathology and could potentially represent a predictive factor of liver damage aggravation. As previously described [26] high serum IL-10 levels may be related to a poor response to IFN treatment in patients with chronic hepatitis C. In this sense, it would have been interesting to investigate other markers such as spleen markers or beta2-microglobulin, although it has been described that neither spleen measurements nor serum beta2-microglobulin levels were able to predict therapeutic response to antiviral therapy [27]. In conclusion of our study, the increase of Tr1 cells, which suppressed protective Th1 responses via their high production of IL-10, could explain the failure of the IFN treatment observed for this patient. In a more general way, the implication of Tr1 in viral persistence and associated hepatic pathologies could have significant implications for our understanding of the role of T cells in immunity to infectious diseases and for the development of new therapies for the control of immune-mediated disorders. 

## Figures and Tables

**Table 1:** Primer sequences used in real-time quantitative RT-PCR assay.

Gene	Primers sequences	Annealing temperature (°C)
hCD4:	5′-GGGAAATCAGGGCTCCTTCTTA 5′-TGGTCCCAAAGGCTTCTTCTT	59
hCD25:	5′-GGGACTGCTCACGTTCATCA 5′-TTCAACATGGTTCCTTCCTTGTAG	59
hCD49b:	5′-CAACGGGTGTGTGTTCTGACA 5′-TCATCACACACAACCACAACATCT	59
hCD18:	5′-ATGCTTGATGACCTCAGGAATGT 5′-ACGGTCTTGTCCACGAAGGA	59
hT1-ST2:	5′-GTGTTTGCCTCAGGCCAACT 5′-TGACATTCGCATATCCAGTCCTA	59
hCCR5:	5′-GTCAAGTCCAATCTATGACATCAATTATT 5′-CGGGCTGCGATTTGCTT	57
hCD8*α*:	5′-CCCTGAGCAACTCCATCATGTAC 5′-GGCGTCGTGGTGGGC	60
hCD8*β*:	5′-TGGCCGCGCAGCTG 5′-CTTGTTGGTTTGCACCTTTATGTATG	55
hCD19:	5′-CTCACCCCCATGGAAGTCAG 5′-CTTGAGGCACTGCAGCACAG	60
hCD11c:	5′-AATTCAGGCGCACGTCAAA 5′-ATCCCTACGGGCCCCATAT	56
hICAMI:	5′-CCCTGATGGGCAGTCAACA 5′-GCAGCGTAGGGTAAGGTTCTTG	60
h*β*-actin:	5′-CACGGCATCGTCACCAACT 5′-AGCCACACGCAGCTCATTG	58
hIL-4:	5′-CACAAGCAGCTGATCCGATTC 5′-TTCCAAGAAGTTTTCCAACGTACTC	59
hIL-2:	5′-ACCAGGATGCTCACATTTAAGTTTTAC 5′-TCCAGAGGTTTGAGTTCTTCTTCTAGA	61
hIL-10:	5′-GAGAACCAAGACCCAGACATCAA 5′-CCACGGCCTTGCTCTTGTT	59
hIL-10R*α*:	5′-CCGAGAGTATGAGATTGCCATTC 5′-CAGATGGTTTCACCTGGACACA	60
hIL-10R*β*:	5′-TGGGAGTCACCTGCTTTTGC 5′-TCCGTCAAGGTAGTATTCATGCA	59
hIFN*γ*:	5′-ATGTAGCGGATAATGGAACTC 5′-GACATTCAAGTCAGTTACC	53
hTGF-*β*1:	5′-CGAGCCTGAGGCCGACTAC 5′-CGGAGCTCTGATGTGTTGAAGA	62
hTGF-*β*R1:	5′-TGACAACGTCAGGTTCTGGCT 5′-AATCGACCTTTGCCAATGCT	57
hTGF-*β*R2:	5′-GCTGCTTCTCCAAAGTCATT 5′-AACAAGTCAGGATTGCTGGTGTT	58
hP-Selectin:	5′-CTGGAACCCCTGAGTCTACCAC 5′-GTCTGTATCTCCATAGCTGCTGAATC	63
h5′G3PDH:	5′-CCATCAATGACCCCTTCATTG 5′-CTTGACGGTGCCATGGAATT	58
